# Suppression of Cancer Progression by MGAT1 shRNA Knockdown

**DOI:** 10.1371/journal.pone.0043721

**Published:** 2012-09-05

**Authors:** Reza Beheshti Zavareh, Mahadeo A. Sukhai, Rose Hurren, Marcela Gronda, Xiaoming Wang, Craig D. Simpson, Neil Maclean, Francis Zih, Troy Ketela, Carol J. Swallow, Jason Moffat, David R. Rose, Harry Schachter, Aaron D. Schimmer, James W. Dennis

**Affiliations:** 1 Ontario Cancer Institute, Princess Margaret Hospital, and Department of Medical Biophysics, University of Toronto, Toronto, Ontario, Canada; 2 Samuel Lunenfeld Research Institute, Mt. Sinai Hospital, Molecular Genetics, University of Toronto, Toronto, Ontario, Canada; 3 Department of Molecular Genetics, Donnelly Centre for Cellular and Biomolecular Research, Toronto, Ontario, Canada; 4 Department of Biology, University of Waterloo, Waterloo, Ontario, Canada; 5 Program in Molecular Structure and Function, Hospital for Sick Children, University of Toronto, Toronto, Ontario, Canada; Queensland University of Technology, Australia

## Abstract

Oncogenic signaling promotes tumor invasion and metastasis, in part, by increasing the expression of tri- and tetra- branched N-glycans. The branched N-glycans bind to galectins forming a multivalent lattice that enhances cell surface residency of growth factor receptors, and focal adhesion turnover. N-acetylglucosaminyltransferase I (MGAT1), the first branching enzyme in the pathway, is required for the addition of all subsequent branches. Here we have introduced MGAT1 shRNA into human HeLa cervical and PC-3-Yellow prostate tumor cells lines, generating cell lines with reduced transcript, enzyme activity and branched N-glycans at the cell surface. MGAT1 knockdown inhibited HeLa cell migration and invasion, but did not alter cell proliferation rates. Swainsonine, an inhibitor of α-mannosidase II immediately downstream of MGAT1, also inhibited cell invasion and was not additive with MGAT1 shRNA, consistent with a common mechanism of action. Focal adhesion and microfilament organization in MGAT1 knockdown cells also indicate a less motile phenotype. *In vivo*, MGAT1 knockdown in the PC-3-Yellow orthotopic prostate cancer xenograft model significantly decreased primary tumor growth and the incidence of lung metastases. Our results demonstrate that blocking MGAT1 is a potential target for anti-cancer therapy.

## Introduction

Metastatic cancers generally have a poor prognosis, and show limited responses to chemotherapy and newer targeted therapies [1]. The redundancy in growth receptors and signaling contributes to invasion and drug resistance, a problem that might be overcome by considering additional levels of feedback regulation. In this regard, receptors are N-glycosylated in the ER and the N-glycans remodeled in the Golgi on route to the cells surface [2]. Oncogenic transformation increases Ets-driven transcription of N-acetylglucosaminyltransferase V encoded by Mgat5, a medial Golgi enzyme that initiates the β1,6GlcNAc antenna in tri- and tetra- branched N-glycans [3]–[5]. Over-expression of Mgat5 in immortalized epithelial cells has been shown to relax growth controls and promote tumorogenesis when the cells are injected into mice [6]. The oncogenic induced expression of Mgat5 and its β1,6GlcNAc tri- and tetra- branched N-glycans products correlate with distant metastasis and reduced survival in human mammary and colon cancers [7], [8]. In mice, tumor progression is delayed in the polyoma-virus middle T transgenic (PyMT) and the Pten^+/−^ models of cancer on an Mgat5 deficient background [9], [10]. Moreover, the Golgi α-mannosidase II inhibitor swainsonine inhibits tumor cell metastasis in mouse models, and was tested with promising results in clinical trials [11], [12].

**Figure 1:**
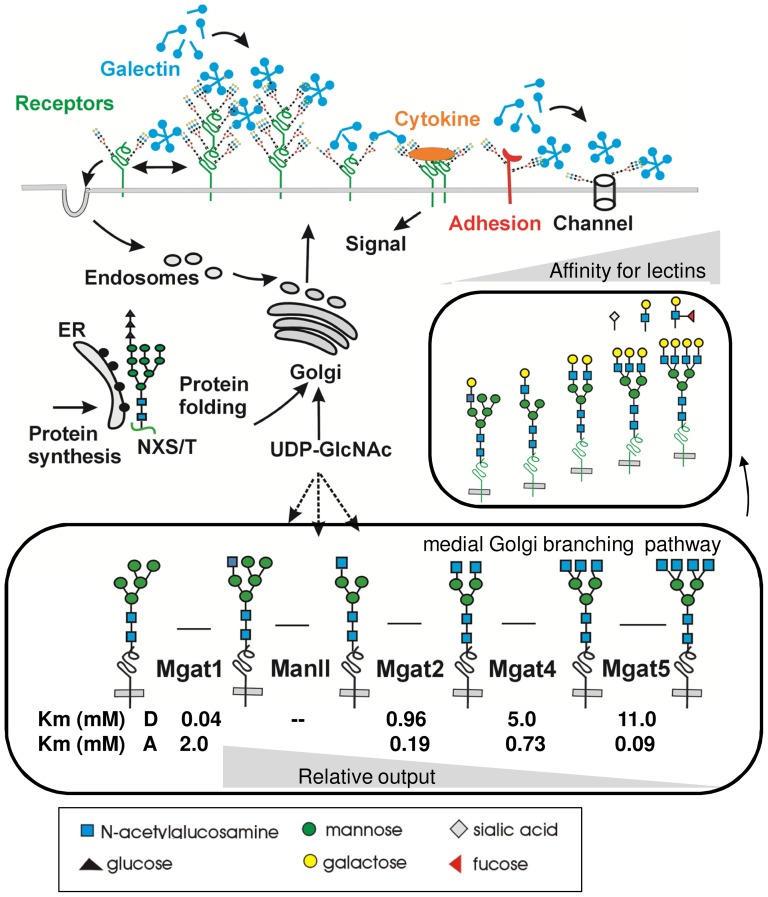
Schematic of *N*-glycan branching and receptor dynamics at the cell surface. Oligosaccharyltransferase (OST) substitutes NXS/T sites of proteins in the rough endoplasmic reticulum (RER), transferring the pre-assembled glycan from Glc_3_Man_9_GlcNAc_2_-pp-dolichol to the Asn. Glycoproteins traffic to the Golgi where the N-glycans are remodeled, and the structural details depend on enzyme expression and UDP-GlcNAc supply. The branching enzymes Mgat1, Mgat2, Mgat4, and Mgat5 differ in Km values for UDP-GlcNAc “D” and for glycoprotein acceptor “A”. The pathway has evolved for pathway ultrasensitivity to UDP-GlcNAc. The epitopes are completed in the trans-Golgi (small box) where efficient substitution with Gal generates the LacNAc epitope, and can be extended by poly-LacNAc. Further extension with galactose generates epitopes for galectin binding, with effects on receptor dynamics, interactions and trafficking.

Galectins bind to the N-glycans on receptors and solute transporters with affinities proportional to branching and the number of N-glycans (NXS/T attachment sites) [13], [14]. Galectin-3 has been shown to cross-link glycoprotein receptors at the cell surface, forming a dynamic lattice that slows trafficking into coated-pit endosomes and caveolin-1 lipid rafts, thereby promoting surface residency and sensitivity to ligands [14], [15]. The lattice is heterogeneous and has been shown to regulate surface retention of EGF, TGF-β and VEGF receptors [14], [16] as well as GLUT-2, -4 glucose transporters and TRPV5 Ca^++^ channel [13], [17], [18] (Figure 1). Branched N-glycans also enhance turnover of substratum and cell adhesions, supporting the epithelial-mesenchymal transition (EMT) in cancer cells [19], [20]. Moreover, Mgat5 transgene expression in mouse skin promotes EMT-like phenotype and wound healing [21]. Mgat5^−/−^ PyMT-mammary tumor cells in culture show reduced surface residency of cytokine receptors, which can be rescued by (i) Mgat5 re-expression, (ii) inhibiting constitutive endocytosis, (iii) depletion of caveolin-1 and (iv) by GlcNAc supplementation to UDP-GlcNAc the common donor for the Mgat enzymes [13], [14]. GlcNAc supplementation in Mgat5^−/−^ cells increases the content of bi- and tri-antennary N-glycans, which rescues affinities for galectin-3 without the Mgat5 product [13]. This suggests redundancy in N-glycan structures and profiles that can support the malignant phenotype.

Mgat1 catalyzes the addition of the first branch, and the product is required for the action of α-mannosidase II and Mgat2, and then Mgat4 and Mgat5 in a catalytically ordered pathway [2] (Figure 1). Low Mgat1 activity limits the output of tri- and tetra-branched end products, but high levels can also do the same by depriving UDP-GlcNAc supply to Mgat4 and Mgat5 enzyme later in the pathway. Indeed, Mgat enzymes display decreasing affinity for UDP-GlcNAc, which results in pathway ultrasensitivity, with a characteristic sigmoidal output of Mgat5 products in response to this shared metabolite [13]. The hexosamine pathway substrates intersect with basic metabolism which is known to undergo extensive change in cancer cells [22]. UDP-GlcNAc synthesis by the hexosamine pathway depends on glucose, glutamine, and acetyl-CoA supply to the hexosamine pathway, as well as GlcNAc salvage. GlcNAc and GlcNAc-p can be elevated at an intermediate stage in prostate cancer progression [23], and may play a role in promoting tumor progression [24]. Therefore, inhibiting branching early in the pathway may be a viable strategy to avoid metabolic compensation.

To begin exploring the role of MGAT1, we generated human tumor cell lines stably expressing MGAT1 shRNA, and achieved ∼70% suppression of branched *N*-glycans and the invasive phenotype, without altering proliferation and viability in cell culture. MGAT1 knockdown decreased growth and metastasis of human prostate cancer cells in a xenograft orthotopic mouse model. Although MGAT1 is required for mouse development beyond day E9.5 [25], [26], our results suggest that partial systemic depletion of MGAT1 in adults may be tolerable and have important anti-cancer effects.

## Materials and Methods

### Cell culture

HeLa human cervical cancer cells (purchased from ATCC) were maintained in Dulbecco's modified Eagle Medium (DMEM). PC-3-Yellow cells (a highly metastatic clone of PC-3 human prostate cancer cell line stably expressing red fluorescent protein (RFP) and green fluorescent protein (GFP) which was a gift from G. Glinsky, Ordway Research Institute, [27]) were maintained in RPMI 1640. All cells were supplemented with 10% fetal bovine serum (FBS) (Hyclone, Logan, UT), antibiotics, cultured in a standard humidified incubator at 37°C in a 5% CO_2_ atmosphere.

### L-PHA binding to cell surface glycans

Cells were seeded in 96-well plates at 500 cells per well. After adhering overnight, cells were fixed with 3.7% formaldehyde and washed. Surface tri- and tetra- branched *N*-glycans were stained with L-PHA (20 µg/mL) conjugated to Alexa Fluor 488 or Alexa Fluor 647 and nuclei were stained with 5 µg/ml of 4′,6-diamidino-2-phenylindole, dilactate (DAPI, dilactate) (Molecular Probes, Eugene, OR, USA). The total intensity of L-PHA staining on each cell was quantified using Cellomics ArrayScan II (Cellomics, Pittsburgh, PA) [13].

### MGAT1 silencing by lentiviral-delivered RNA interference

Construction of hairpin-pLKO.1 vectors (carrying a puromycin antibiotic resistance gene) containing short hairpin RNA (shRNA) sequences and production of shRNA viruses have been described in detail [28]. The shRNAs targeting the MGAT1 coding sequences are as follows: *MGAT1*-sh1 (NM_002406), 5′-CCCTGAGATCTCAAGAACGAT-3′; and *MGAT1*-sh2 (NM_002406), 5′-GCACCTCAAGTTTATCAAGCT-3′. The control shRNA coding sequences are as follows: RFP, 5′-CTACAAGACCGACATCAAGCT-3′ and LacZ, 5′-CCGTCATAGCGATAACGAGTT-3′. Lentiviral infections were done essentially as described previously [28]. Briefly, adherent cells were treated with 0.5 mL of the virus followed by overnight incubation (37°C, 5% CO_2_) without removing the virus. The next day, viral medium was replaced with fresh medium containing puromycin (1 µg/mL) to select a population of resistant cells.

### Reverse-transcriptase real-time PCR

First-strand cDNA was synthesized from 1 µg of DNase-treated total cellular RNA using random primers and SuperScript II reverse transcriptase (Invitrogen) according to the manufacturer's protocol. Real-time PCR assays were performed in triplicate with 5 ng of RNA equivalent cDNA, SYBR Green PCR Master mix (Applied Biosystems, Foster City, CA, USA), and 400 nM of gene-specific primers. Reactions were processed and analyzed on an ABI 7900 Sequence Detection System (Applied Biosystems). Forward/reverse PCR primer pairs for human cDNAs were as follows: human GlcNAc-TI: Forward 5′- CGGAGCAGGCCAAGTTC-3′, Reverse 5′- CCTTGCCCGCAGTCCTA-3′,18S: Forward 5′- AGG AAT TGA CGG AAG GGC AC-3′, Reverse 5′-GGA CAT CTA AGG GCA TCA CA-3′. Relative mRNA expression was determined using the ΔΔCT method as described.

### GlcNAc-transferase activities

The enzyme activity of MGAT1was measured using a synthetic receptors as previously described [29]. Briefly, cell lysates were incubated with MGAT1 acceptor Manα(1,3) [Manα(1,6)]Glcβ1-O-(CH_2_)_7_CH_3_(Toronto Research Chemicals, Toronto, ON), in a solution containing 0.5 mM UDP-[6^3^H]GlcNAc(44400 dpm/nmol), 125 mM MES (pH 6.5), 50 mM GlcNAc, 1 mM UDP-GlcNAc, 0.8 mM AMP and 10 mM MnCl2. After incubation, the reaction was stopped by adding ice-cold H_2_O. Transfer of [6^3^H]GlcNAc to the acceptor was quantified by liquid scintillation counting.

### Migration and invasion assays

Invasion and migration assays in HeLa cells were performed as previously described [30]. Briefly HeLa cells (2×10^5^) were harvested and seeded in uncoated invasion chambers for migration assay or in BioCoat Matrigel Invasion Chambers (BD Biosciences, Mississauga, ON) for invasion assays. For both the migration and invasion assays, growth medium containing 10% FBS was used as a chemoattractant in the bottom well. Following 48 hours of incubation, cells that had migrated or invaded the lower surface of the membrane were stained with Diff-Quik Stain (BD Biosciences). The number of migrating or invading cells were imaged and counted using the Aperio ScanScope CS whole slide Scanner (AperioTechnologies, Vista, CA) and Image-Pro Plus Software (version 4.5; Media Cybernetics Inc., Silversprings, MD). In order to test the effect of simultaneous MGAT1 inhibition and swainsonine treatment, HeLa cells were treated with 2 µM swainsonine for 72 hours prior to the migration and invasion assay as described above.

To assess invasion and migration in PC-3-Yellow cells, cells were serum deprived over night and seeded in the upper chamber of 16-well CIM-Plate (Roche Applied Sciences) for migration assays or Matrigel (BD Biosciences, Mississauga, ON) coated plate for invasion assays. The lower chamber containing 20% FBS as chemo-attractant, and the plates were placed in the Roche xCELLigence Real-Time Cell Analyzer (RTCA) DP platform (Roche Applied Sciences). Real-time measurements were done every five minutes for 36 hours. The RTCA Analyzer measures electrical resistance of cells that move to lower surface of the membrane, and compares well with the cell-count method above.

### Immunocytochemistry

Cells were seeded on fibronectin-coated cover slips (Sigma-Aldrich, Oakville, ON). After adhering overnight, cells were fixed with 2% paraformaldehyde for 10 minutes, followed by permeabilization using 0.2% Triton-X-100. After blocking by 5% BSA for 1 hour, cells were incubated overnight at 4°C with mouse anti-β1 integrin (1∶200, Millipore) or anti-phospho-paxillin (pY31) antibody (1∶400, Epitomics). Cover slips were washed with PBS and then incubated with donkey anti-mouse IgG-Cy3 conjugated secondary antibody (1∶200, Millipore) for β1 integrin or anti-rabbit IgG-FITC conjugated antibody (1∶500, Millipore) for paxillin. Staining for actin was done by phalloidin conjugated to tetramethylrhodamine B isothiocyanate (TRITC, Sigma-Aldrich) for 10 minutes. After washing with PBS, cover slips were mounted on slides using fluorescent mounting medium and imaged using the 40× lens of the Zeiss LSM700 confocal microscope (Carl Zeiss MicroImaging GmbH, Jena, Germany).

### In vivo model of prostate cancer metastasis

Distant tumor formation was evaluated *in vivo* as previously described [31]. Briefly, PC-3-Yellow cells stably expressing RFP with and without MGAT1 knockdown, were injected orthotopically into the prostates of sublethally irradiated (3.5 Gy) SCID mice. Mice injected with tumor cells were maintained for 4 weeks after injection, at which time, the animals were sacrificed via cervical dislocation for complete examination. Red fluorescent tumors were detected via whole body imaging and whole organ imaging using a Leica MZ FLIII fluorescent stereomicroscope with a 100 W mercury lamp, a 560/40 excitation filter, and a 610 long-pass emission filter. Images were acquired using an Olympus DP70 digital camera at 0.8× magnification and analyzed using Image Pro Plus 6.0 (Media Cybernetics). A single common threshold was applied to identify and measure fluorescence in each organ as the number of fluorescent spots per lung lobe. Mice were obtained from an in-house breeding program and housed in laminar-flow cage racks under standardized environmental conditions with *ad libitum* access to food and water. All experiments were approved by the local institutional ethics review board (University Health Network-Ontario Cancer Institute Animal Care and Use committee) and were performed according to the regulations of the Canadian Council on Animal Care. All efforts were made to minimize suffering.

## Results and Discussion

### MGAT1 shRNAi reduces N-glycan branching, cell migration and invasion

**Figure 2:**
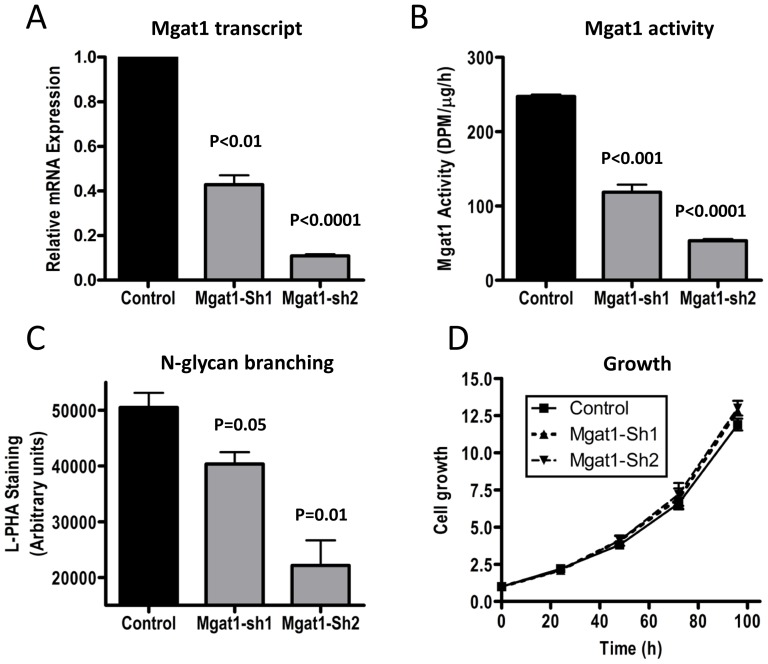
shRNA MGAT1 suppresses N-glycan branching. **A**) HeLa cells were infected with lentiviral vectors targeting MGAT1(shRNA1 or shRNA2) or the control shRNA sequences. Stable cell populations were selected by the addition of puromycin (1 µg/mL). **A**)MGAT1 mRNA were measured by qRT-PCR, **B**) MGAT1 enzyme activity, **C**) L-PHA reactive surface N-glycans by Array scan microscope, **D**) Proliferation over 4 days Data represent the mean ± SD relative expression of mRNA relative to control sequence (n = 3 independent experiments performed in triplicate).

To assess the cell autonomous effects of MGAT1 depletion on malignant cell growth and invasion, HeLa human cervical cancer cells were infected with lentivirus shRNA vectors targeting MGAT1 or control sequences, and stable cell populations were selected with puromycin. Target knockdown using two independent shRNA sequences was confirmed by qRT-PCR (Figure 2A). shRNA1 and shRNA2 also reduced MGAT1 enzymatic activity and decreased L-PHA cell-surface staining in proportion to the depletion of mRNA and enzyme activity (Figure 2B, C). L-PHA binds to products down-stream of MGAT1, the β1,6GlcNAc tri- and tetra- branched N-glycans [32]. MGAT1 knockdown did not alter cell viability or proliferation (Figure 2D and data not shown).

**Figure 3:**
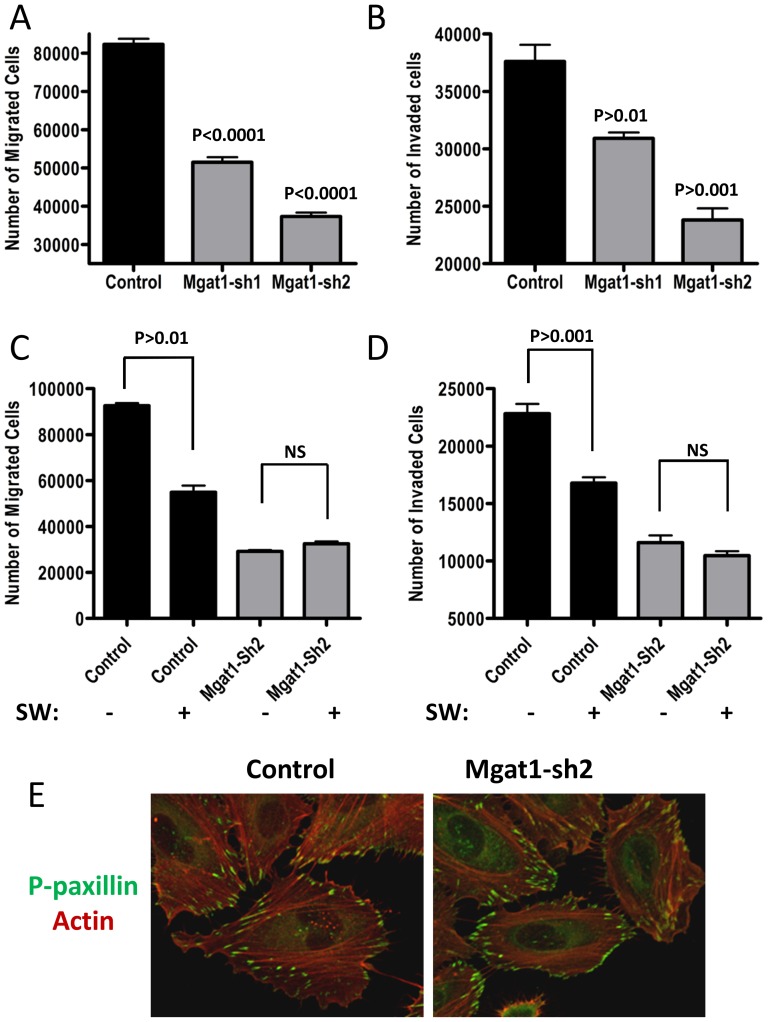
MGAT1 shRNA inhibits HeLa cell migration and invasion *in vitro*. **A**) HeLa cells were plated into chambers with 8-µm pores and 10% FBS was used as a chemoattractant. **B**) HeLa cells were plated into invasion chambers with Matrigel. After 48 h, cells that had migrated through the pores were fixed, stained and counted automatically. C) migration and D) invasion assays with or without pretreatment for 72 h with 2 µM swainsonine (SW) for 72 hours prior. The mean number of migrated cells ± SD of 3 independent experiments performed in triplicate were graphed. (**E**) Cell morphology by staining with phospho-paxillin and TRITC-conjugated phalloidin for F-actin, shown as a merged image.

To evaluate the effects of MGAT1 shRNA2 knockdown on HeLa cell migration and invasion, the cells were seeded into chambers on porous filters in serum-free medium, and 10% FBS was placed in the lower chamber as a chemo-attractant. Cell migration into the bottom chamber was measured 48 h later. Similar studies were conducted using Matrigel-coated filters to measure cell invasion though a barrier. Knockdown of MGAT1 decreased cell migration and invasion, in proportion to MGAT1 depletion (Figure 3A, 3B). Blocking the pathway one step downstream of MGAT1, at α–mannosidase II, using swainsonine also inhibited invasion as previously reported [33], [34]. Swainsonine treatment alone inhibited migration and invasion, but to a lesser degree than shRNA2. This may be due to the paralog α–mannosidase IIx, which is less sensitive to swainsonine. Importantly, swainsonine had no additional effects in MGAT1 shRNA2 cells, suggesting that optimal suppression of migration and invasion by targeting the branching pathway is observed with ∼70% depletion of MGAT1 (Figure 3C, 3D).

Cell migration rates are partly dependent on α5β1 integrin receptor contacts with substratum fibronectin, which stimulate focal adhesions signaling, turnover and propulsion [35]. The branched N-glycans products of MGAT5 are present on α5β1, among other receptors and cell surface proteins, and have been shown to promote α5β1 activation, signaling, and tumor cell migration [19]. Integrin activation results in phosphorylation of paxillin by FAK and Src, leading to F-actin remodeling and cell motility [36]. In HeLa cells with MGAT1 knockdown, the F-actin stress fibers were smaller in diameter, less convergent on cell projections, and tended to circumscribe the cell (Figure 3E). Staining of p-paxillin revealed smaller and more clusters at the edge of cellular projections. In contrast, control HeLa cells had prominent and long stress fibers that project into pseudopodia ending with larger phospho-paxillin staining focal adhesions, characteristic of a more motile phenotype than the MGAT1 knockdown cells.

### MGAT1 knockdown decreases tumor growth and metastasis *in vivo*

**Figure 4:**
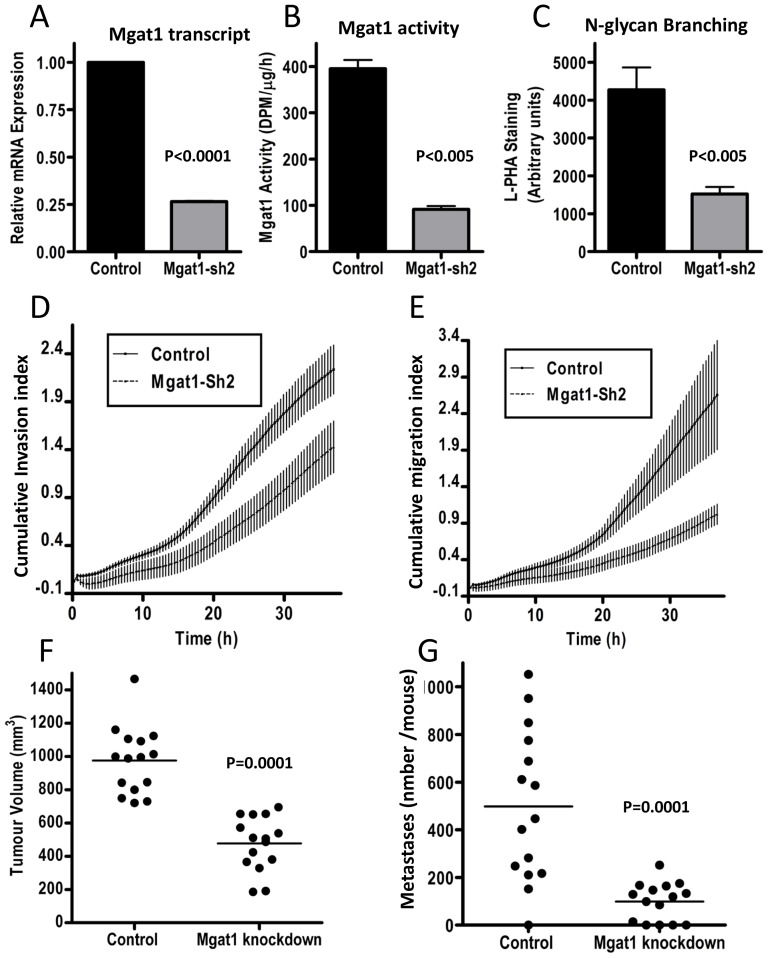
MGAT1 knockdown decreases tumor migration, and invasion in prostate cancer cells. PC-3-Yellow cells with MGAT1-shRNA2 or the control shRNA sequences were assessed for **A**) MGAT1 mRNA levels by qRT-PCR. **B**)MGAT1 enzyme activity, **C**) L-PHA reactive surface N-glycans by Arrayscan microscope, **D**) invasion through a Matrigel barrier and migration (**E**) using the xCELLigence Real-Time Cell Analyzer. Data represents mean ±SD cell index (3 independent experiments with quadruplicates). (**F**) Tumor size and (**G**) metastasis in mice injected with PC-3-Yellow cells with MGAT1-shRNA2 or the control shRNA sequences. The mice were injected orthotopically, with 0.5×10^6^ cell per mouse, into the prostate of the sub-lethally irradiated SCID mice. Four weeks after injection, mice were sacrificed and their organs were imaged using a fluorescent microscope. The numbers of metastatic nodules in all five lobes of the lungs were quantified using image analysis software. Each point represents one mouse.

To assess the effects of MGAT1 knockdown in an animal model of cancer metastasis, we used the human PC-3-Yellow prostate cancer cells, a well-established xenograft model. PC-3-Yellow cells were infected with the lentivirus containing MGAT1 shRNA2 or control sequences, and stable cell populations were selected as above. MGAT1 mRNA, enzymatic activity, and cell surface branching were all depleted by ∼70% (Figure 4A–C). MGAT1 knockdown decreased both cell migration and cell invasion in an in vitro and in vivo model (Figure 4D,E). MGAT1 knockdown did not alter the growth and viability of PC-3-Yellow cells (Data not shown). Thus, the *in vitro* phenotypes were remarkably similar to the effects of MGAT1 knockdown in HeLa cells. To assess the effects of MGAT1 knockdown on tumor metastasis, control or MGAT1 knockdown PC-3-Yellow prostate cancer cells were injected orthotopically into the prostate glands of sub-lethally irradiated SCID mice (n = 15 per group). Four weeks after injection, mice were sacrificed and distant tumor formation in the organs was imaged by fluorescent microscopy. Tumors developed in the prostate in all mice injected with MGAT1 knockdown and control RFP-labeled PC-3-Yellow cells. In mice injected with control cells, tumor formation was detected at clinically relevant sites of metastases, including lung, lymph nodes and liver. However, tumors volume was reduced and fewer distant lung metastases were observed in mice injected with MGAT1 knockdown cells (Figure 4F,G). Overall, more of the mice in the MGAT1 knockdown group were free of metastases. Thus, taken together, MGAT1 knockdown inhibits distant tumor formation *in vivo*.

In summary, ∼70% MGAT1 knockdown suppress N-glycan branching and the invasive phenotype in HeLa and PC-3-Yellow cells. Importantly, MGAT1 knockdown decreased growth and metastasis of PC-3-Yellow cells in an orthotopic prostate cancer xenograft model. Each of the N-glycan branches is an epitope for galectins, and cumulatively they can support growth signaling at the cell surface. Compensation and rescue of the galectin lattice by epitope transfer between branches has been observed in Mgat5^−/−^ cancer cells [13] and in Mgat4^−/−^ mouse tissues [37]. Oncogenic signaling in cancer cells up-regulates the transcription and activities of MGAT4 and Mgat5 [4], [5], [38] as well as hexosamine pathway intermediates [23], but targeting MGAT1 avoids these mechanisms of rescue. Therefore, MGAT1 may be useful targets in cancer treatment to block metastasis as well as tumor growth.
